# Shallow Whole Genome Sequencing for the Assembly of Complete Chloroplast Genome Sequence of *Arachis hypogaea* L.

**DOI:** 10.3389/fpls.2016.01106

**Published:** 2016-07-27

**Authors:** Sudheesh K. Prabhudas, Sowjanya Prayaga, Parani Madasamy, Purushothaman Natarajan

**Affiliations:** Department of Genetic Engineering, SRM UniversityKattankulathur, India

**Keywords:** complete chloroplast genome, *Arachis hypogaea*, peanut, groundnut, *de novo* assembly, illumina

## Introduction

The chloroplast (CP) is a plant organelle originated from cyanobacteria through symbiosis and had become an important component of the plant cell. It is the reaction center for the photosynthesis and also for several steps in the biosynthetic pathways of fatty acids, vitamins, pigments and amino acids. The CP genome is highly conserved in land plants (Raubeson and Jansen, 2005). The CP genome is circular and exhibits a quadripartite genome structure consisting of a large single copy region (LSC) and a small single copy region (SSC), separated by a pair of inverted repeats (IRs) with a few exceptions where loss of an IR or the SSC was observed. The size of the CP genome varies from 19 to 217 Kb in land plants, and the IRs are usually 20–26 kb in length (http://www.ncbi.nlm.nih.gov/genome/organelle/). Lack of recombination makes the CP genome an ideal target for phylogenetic studies (Ravi et al., 2008; Wu and Ge, 2012).

*Arachis hypogaea* L. also known as groundnut is an herbaceous plant belonging to the Fabaceae family. It has an allotetraploid genome (AABB; 2n = 4x = 40) with a size of about 2.8 Gb. There have been many speculations regarding the ancestors of A and B subgenomes of *A. hypogaea* and proved to have originated through a hybridization event between *Arachis ipaensis* L. (B subgenome) and *Arachis duranensis* L. (A subgenome) (Kochert et al., 1996; David et al., 2016). It is one of the major edible oilseed crops in the world, and India is the second largest producer accounting for about 15% of the world production (FAOSTAT, 2015). Kernels of *A. hypogaea* L. contains 43–50% oil and 23–26% proteins. The oil comprises majorly of palmitic acid (16:0), stearic acid (18:0), oleic acid (18:1), linoleic acid (18:2), arachidic acid (20:0), eicosenoic acid (20:1), behemic acid (22:0), and lignoseric acid (24:0) along with trace amounts of palmitoleic acid (16:1). The mono and poly-unsaturated fatty acids, oleic acid and linoleic acid constitute about 75% of the total oil content (Shiv, 1982). Many attempts have successfully been made to improve the crop yield, drought resistance, disease resistance and other characteristics of *A. hypogaea* L. using classical breeding as well as genetic engineering using nuclear transformation. Chloroplast transformation by homologous recombination for producing transgenic plants is also possible due to the presence of candidate loci on the CP genome. Additionally, Genetic engineering of chloroplast genome when compared to nuclear transformation is environment-friendly; it minimizes the pleiotropic effects along with containment of the foreign genes (Daniell et al., 2005). Hence, the availability of the complete chloroplast genome of *A. hypogaea* L. will be an invaluable resource for designing and evaluating efficient chloroplast transformation experiments.

## Materials and methods

### Plant material and genome sequencing

The seeds of *A. hypogaea* L. Co7 variety were obtained from Tamilnadu Agricultural University, Coimbatore, India. The plants were grown in the green house facility at SRM University, Kattankulathur, India. Leaves from 1-month old plant were used for total genomic DNA isolation using DNeasy Plant Mini Kit (Qiagen, Germany). A paired-end library with an average insert size of about 400 bp was constructed as per the manufacturer's protocol (Illumina Inc., USA). The library quality was assessed on CaliperLabChip GX using High Sensitivity Assay Kit (Caliber, USA). It was then hybridized on a flow cell for generating clonal clusters on cBOT using Truseq PE Cluster Kit v3-cBot-HS (Illumina Inc., USA). Sequencing by synthesis was performed on Illumina Hiseq 2500 using Truseq v3-HS kit to generate 100 bp paired end reads (Illumina Inc., USA).

### Genome assembly and validation

The per base quality of the raw paired-end reads (51,650,486) of 100 bp was assessed by FastQC v0.11.2 (Andrews, 2010). The adapter trimming and quality filtering was done using Cutadapt v1.7.1 (Martin, 2011) and Sickle v1.33 (Joshi and Fass, 2011) tools respectively. A phred score of 20 was used for quality filtering. The quality filtered paired-end reads (49,299,308) were subjected to *de novo* assembly using three different *de novo* assemblers such as Velvet v1.2.10 (Zerbino and Birney, 2008), SOAPdenovo v2.04 (Luo et al., 2012) and Edena v3.131028 (Hernandez et al., 2008). The assembled contigs were pooled and ordered against the complete CP genome of closest relative *Acacia ligulata* L. as the reference using Mauve v2.3.1 tool (Darling et al., 2010; Williams et al., 2015). The gaps in the genome were filled by manual alignment of paired-end reads using overlapping method (Natarajan and Parani, 2015) and primer walking (Sanger sequencing method). Validation of the junctions between the single copy regions and the inverted repeats was done by Sanger sequencing using specific primers. The filtered reads were mapped against the assembled CP genome of *A*. *hypogaea* L. to calculate the genome coverage. The complete CP genome of *A. hypogaea* L. was annotated using DOGMA (Wyman et al., 2004).

## Results and discussion

The size of the complete CP genome of *A. hypogaea* L. was found to be 156,391 bp. The genome coverage was calculated to be 2122x with 3,863,475 quality filtered reads mapped to the assembled CP genome. The CP genome exhibited a quadripartite structure consisting of LSC and SSC regions of 85,946 bp and 18,797 bp respectively, with a pair of inverted repeats (IRa and IRb) of 25,824 bp each separating them. The overall GC content of the complete chloroplast genome was 36.4% and the individual GC content for LSC, SSC, and IRs was 33.8%, 30.2%, and 42.8% respectively. A total of 110 genes were annotated including 76 protein coding genes, 30 tRNA genes, and 4 rRNA genes. Six of the protein coding genes and the 3' exon of rps12 are duplicated in the IR regions. Six of the tRNA genes and four of the rRNA genes are also duplicated in the IR regions. The presence of one or two introns were identified in the 13 genes, which includes 8 protein coding genes and 5 tRNAgenes (Table 1). The complete CP genome sequence of *A. hypogaea* that is reported here for the first time will be an invaluable resource for designing and evaluating efficient chloroplast transformation experiments and to improve the desired traits.

**Table 1:** **List of genes found in the ***A. hypogaea*** L. chloroplast genome**.

**S.No**	**Group of genes**	**Gene names**
1	ATP synthase	atpA, atpB, atpE, atpF^*^, *atpH, atpI*
2	Cytochrome b/f complex	*petA, petB, petD, petG, petL, petN*
3	NADH dehydrogenase	ndhA^*^, *ndhC, ndhD, ndhE, ndhF, ndhG, ndhH, ndhI, ndhJ*
4	Photosystem I	*psaA, psaB, psaC, psaI, psaJ*
5	Photosystem II	*psbA, psbB, psbC, psbD, psbE, psbF, psbH, psbI, psbJ, psbK, psbL, psbM, psbN, psbT, psbZ*
6	Proteins of unknown function	ycf1, ycf2, ycf3^**^, ycf4, orf42, ycf68^*^
7	Ribosomal proteins (SSU)	*rps2, rps*3, *rps4, rps7, rps8, rps11, rps12^#^, rps14, rps15*, rps18, *rps19*
8	Ribosomal proteins (LSU)	rpl2^*^, *rpl14, rpl16, rpl20, rpl23, rpl32, rpl33, rpl36*
9	Ribosomal RNAs	*rrn4.5, rrn5, rrn16, rrn23*
10	RNA polymerase	rpoA, rpoB, rpoC1^*^, *rpoC2*
11	Other genes	accD, ccsA, cemA, clpP^**^, matK, rbcL
12	Transfer RNAs	*trnA*-UGC^*^, *trnC*-GCA, *trnD*-GUC, *trnE*-UUC, *trnF*-GAA, *trnfM*-CAU, *trnG*-UCC, *trnH*-GUG, *trnI*-CAU, *trnI*-GAU^*^, *trnK*-UUU^*^, *trnL*-CAA, *trnL*-UAA^*^, *trnL*-UAG, *trnM*-CAU, *trnN*-GUU, *trnP*-GGG, *trnP*-UGG, *trnQ*-UUG, *trnR*-ACG, *trnR*-UCU, *trnS*-GCU, *trnS*-GGA, *trnS*-UGA, *trnT*-GGU, *trnT*-UGU, *trnV*-GAC, *trnV*-UAC^*^, *trnW*-CCA, *trnY*-GUA

* Contains one intron

** Contains two introns

# Exhibits trans-splicing.

## Deposited data and information to the user

The complete data from the current study was submitted at NCBI under the BioProject ID PRJNA314013 and BioSample ID SAMN04527043. The assembled complete chloroplast genome sequence was submitted to NCBI Genbank with an accession number KX257487 (http://www.ncbi.nlm.nih.gov/nuccore/KX257487). The raw reads in compressed FASTQ were submitted to SRA database at NCBI under the accession number SRP076091 (http://www.ncbi.nlm.nih.gov/sra/SRP076091). Users can download and reuse the data for research purpose only with an acknowledgement to us and quoting this paper as reference to the data.

## Author contributions

PN conceived the study and acquired the funding; SKP and SP performed the genome assembly and analysis; SKP, PN, and PM drafted the manuscript. All authors approved the final manuscript.

## Funding

The project was funded by Department of Biotechnology (DBT), Government of India, under the Rapid Grant for Young Investigator (RGYI) scheme (BT/PR6394/GBD/27/422/2012).

### Conflict of interest statement

The authors declare that the research was conducted in the absence of any commercial or financial relationships that could be construed as a potential conflict of interest.
